# Fully resolved genome assembly of a *Macrococcus bovicus* isolated from a human skin infection

**DOI:** 10.1128/mra.00045-25

**Published:** 2025-03-25

**Authors:** Javier E. Fernandez, Alexandra Collaud, Géraldine Jost, Vincent Perreten, Nadia Liassine

**Affiliations:** 1 Division of Molecular Bacterial Epidemiology and Infectious Diseases, Institute of Veterinary Bacteriology, Vetsuisse Faculty, University of Bern 30398, Bern, Switzerland; 2 Dianalabs 197802 , Geneva, Switzerland; Department of Biological Sciences, Wellesley College, Wellesley, Massachusetts, USA

**Keywords:** opportunistic infections

## Abstract

The complete circular genome of *Macrococcus bovicus* LI0213 isolated from a human skin lesion was obtained using a hybrid assembly of Nanopore and Illumina reads. The genome consisting of a 2,082,488-bp chromosome and three plasmids, contains phage-related sequences and represents the first fully resolved genome of *M. bovicus* from human origin.

## ANNOUNCEMENT

Members of the *Macrococcus* genus are skin flora inhabitants of mammals and are rarely associated with infections (1). *Macrococcus bovicus* was originally detected in cattle and horses (2), and an association with infection has not yet been reported. The only *M. bovicus* genome available in the NCBI database is the incomplete draft of the type strain ATCC 51825^T^ (accession number: NZ_SCWF00000000.1) (3). We present the complete circular genome of a human infection-associated *M. bovicus*.

*M. bovicus* LI0213 was isolated in May 2019 in Switzerland from an erythematous lesion on the back of a 47-year-old female outpatient. The infected skin material was collected using an eSwab (Copan), which was streaked on Colombia blood agar (CBA) and chocolate blood agar with PolyVitex (bioMérieux) and subsequently incubated at 37°C for 72 hours. Cultures revealed >50 colonies of *M. bovicus*, which were identified by 16S rRNA gene Sanger sequencing as this bacterium did not belong to the MALDI-TOF MS Bruker MBT 7854 MSP Library. LI0213 was cryopreserved in our collections.

Genomic DNA was extracted from a lawn of colonies grown on CBA at 37°C overnight using the QIAGEN DNeasy Blood & Tissue Kit and purified with CleanNGS magnetic beads (CleanNA). DNA was sequenced using the Nextera DNA Flex Library Prep Kit with IDT Illumina DNA/RNA UD Indexes on an Illumina NovaSeq 6000 system (2 × 150-bp paired-end) (NGS Platform, University of Bern) and with the MinION MK1b device on a R9.4.1 SpotON flow cell using the 1D ligation kit (SQK-LSK109) (Oxford Nanopore Technology, ONT). ONT reads were basecalled using Guppy v4.4.1 (4). Raw Illumina reads were quality-controlled, filtered, and trimmed using Prinseq v0.6 (5). The number of reads and coverage were calculated with NanoStat v1.6.0 (6) for ONT and with fastp v0.24.0 (7) and samtools v1.21 for Illumina (8) (Table 1). The LI0213 genome was hybrid-assembled, circularized, and rotated to DnaA using Unicycler v0.4.8 (5). The resulting genome was annotated using PGAP v6.5. Default parameters were used for all software.

**TABLE 1:** Sequencing statistics and genomic characteristics of *Macrococcus bovicus* strain LI0213 isolated from a human skin infection

Parameters	Genomic features
Genome	Size in bp and GenBank acc. no.
Chromosome	2,082,488 (CP128470)
Plasmids	
pLI0213a (75 CDS)	66,682 (CP128469)
pLI0213b (5 CDS)	4,933 (CP128468)
pLI0213c (4 CDS)	2,933 (CP128467)
GC content (%)	44.5
Illumina statistics	
Read length	151
Number of reads (paired, 2X)	5,425,163
Mean depth	705X
ONT statistics	
N50 (bp)	25.68
Mean read length (bp)	8,449
Number of reads	30,604
Total bases	258,575,387
Coverage	123X
Total number of	
CDSs	2,183
rRNAs	16
tRNAs	62
ncRNAs	4
Pseudogenes	14
CRISPR arrays	1
Integrated elements	Size in bp and position
Prophage phLI0213 (64 CDSs)	42,841 (490,469–532,976)
PICI_LI0213_ (18 CDSs)	13,944 (80,737–94,681)

The assembly of LI0213 consisted of a circular chromosome and three circular plasmids. Species identification of *M. bovicus* was confirmed by an orthologous average nucleotide identity (ANI) value of 98.26% obtained between the genomes of LI0213 and ATCC 51825^T^ (NZ_SCWF00000000.1) using OrthoANIu v0.6.0 (http://www.ezbiocloud.net/tools/ani) (4). GC content, number of predicted protein-coding sequences, pseudogenes, RNA-encoding genes, and CRISPR array are provided in Table 1.

**Fig 1:**
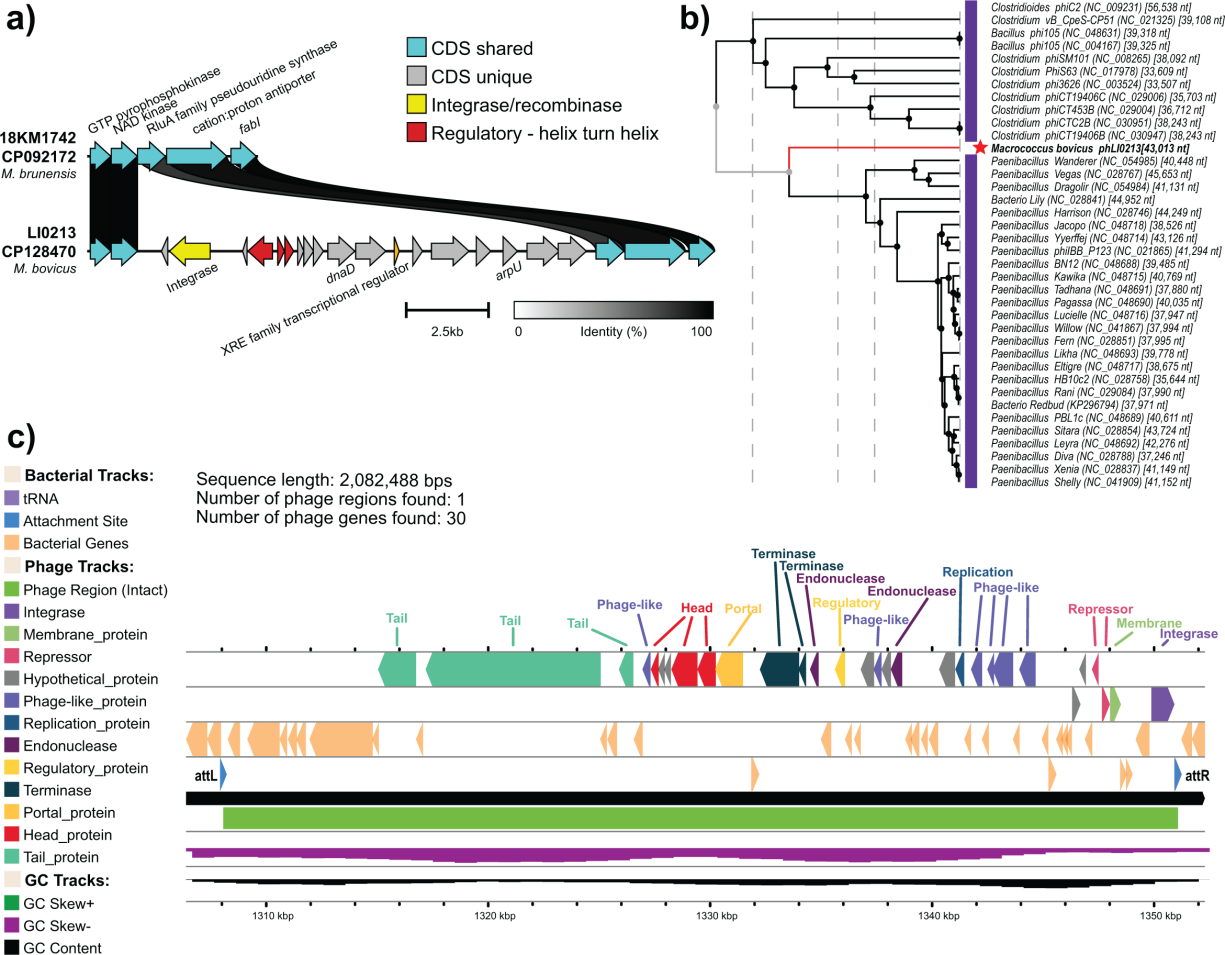
Graphical representation of *Macrococcus bovicus* LI0213 putative prophage phLI0213 and PICI_LI0213_. a) PICI_LI0213_ was identified by manual pairwise comparison of gene clusters of LI0213 and *Macrococcus brunensis* strain 18KM1742. b) Zoomed view of the Virus Proteomic Tree (ViPTree server v4.0 (12)) showing prophage phLI0213 and the closest related phages. Bacterial host, phage name, GenBank accession number, and size are shown as tip labels. c) Graphical representation of phLI0213 as depicted by PHASTEST (10) with attachment sites attL/attR: CCCTCCCAGGACGCTA.

An intact prophage and a putative phage inducible chromosomal island (PICI) were identified in the chromosome of LI0213 (Table 1) (Fig. 1). The PICI_LI0213_ was identified by manual comparison to related strains (Fig. 1A); it remained undetectable by specialized tools (9). The prophage phLI0213 was flanked with attachment sites (att), as identified by PHASTEST (score of 130) (https://phastest.ca) (10) and PhageBoost (11) (Fig. 1C). The tBLASTx-based proteomic tree generated with ViPTree v4.0 (12) placed phLI0213 next to a cluster of *Paenibacillus* spp. phages (Fig. 1B). Virfam (http://biodev.extra.cea.fr/virfam) classified phLI0213 among phages possessing a type 1 neck structural organization and belonging to cluster 2 (Fig. 1C), which is strictly composed of siphophages that infect Firmicutes (13).

## Data Availability

The genome sequence of *M. bovicus* strain LI0213 has been deposited in GenBank under accession numbers CP128470 (chromosome including prophage phLI0213), CP128469 (pLI0213a), CP128468 (pLI0213b), and CP128467 (pLI0213c). The BioProject and BioSample accession numbers are PRJNA984185 and SAMN35742781, respectively. The raw reads were deposited in the SRA database under accession numbers SRX20686773 (ONT) and SRX20686774 (Illumina).
